# Twins methods quantitatively explore the genetic impact on children and adolescents brain gray matter volume

**DOI:** 10.1038/s41598-017-03962-9

**Published:** 2017-07-13

**Authors:** Xiao-Lu Chen, Xiao-Wei Zhang, Xiao Hou, Xiao Li, Xing-Shun Ma, Xiao-Mei Hu, Hua-Qing Meng, Qian He, Lian-Sheng Zhao, Ying-Cheng Wang, Yi-Xiao Fu, Tao Li

**Affiliations:** 1 The First Branch, the First Affiliated Hospital of Chongqing Medical University, Chongqing, 400015 China; 2 Mental Health Center, the First Affiliated Hospital of Chongqing Medical University, Chongqing, 400016 China; 3 Department of Psychiatry and the Centre for Genomic Sciences, the University of Hong Kong, Pok Fu Lam, Hong Kong; 4 Department of Clinical Medicine, Chongqing Medical and Pharmaceutical College, Chongqing, 401331 China; 5 The Second Department of Neurology, The First Hospital of Yulin, Yulin, 719000 Shanxi China; 6 Department of abdominal tumor, Affiliated Hospital of Zunyi Medical College, Zunyi, 563000 China; 7 Department of Radiology, the First Affiliated Hospital of Chongqing Medical University, Chongqing, 400016 China; 8 Mental Health Centre and Psychiatric Laboratory, West China Brain Research Centre, State Key Laboratory of Biotherapy, West China Hospital, Sichuan University, Chengdu, 610041 Sichuan China

## Abstract

The gray matter volumes of 58 pairs of twins ranging in age from 12 to 18 were measured by MRI to explore the genetic and environmental impacts on gray matter volume in twin children and adolescents. By means of A/C/E structural equation modeling, it was found that the gray matter volume in children and adolescents was jointly affected by genetic (A: 0.89) and environmental factors while genetic factors play a greater role. The gray matter volume in frontal lobe, parietal lobe, occipital lobe and lateral temporal lobe was mainly affected by genetics (A: 0.7–0.89), where as the gray matter volume in medial temporal lobe and cingulate cortex was affected by both genetics and environment.

## Introduction

Physiological function and pathological changes have always been the focus of brain research while the brain structure serves as the cornerstone that influences the brain function and disease occurrence. In order to explore brain function and pathological changes, it is critical to understand the brain structure and the process of its development, i.e. the effect of genetics and environment^1^. Brain of child and adolescent is considered to be the optimum research object due to its rapid development.

Twins provide an excellent platform for the investigation of genetic and environmental impact on certain phenotypes. Monozygotic twins carry identical genome, while Dizygotic twins carry genome approximately 50% in common. Therefore study on twins can respectively the genetic and environmental impacts on certain brain areas^2, 3^. Besides, the application of Brain MRI in brain structure research leads a rapid advancement in the field. Studies have shown that brain MRI has played a significant role in observing the whole brain volume and even cortex volume changes^4^, making the quantification of genetic and environmental impact on brain volume becomes possible in combination with twin study.

Most of foreign brain MRI studies focus on the genetic and environmental impact on the whole brain volume in adults, with their results showing that the heritability of the whole brain volume in adults ranges from 0.7–0.9^5^. Studies with a focus on the heritability of gray matter volume are comparatively uncommon. Geschwind *et al*.^6^ found that the heritability of gray matter volume in adult occipital lobe is about 0.28, while the rest region of adult brain is approximately 0.4–0.56. Hulshoff POL *et al*.^7^ identified a high heritability in cortex, left occipital and left posterior cingulate in adults. However, researches on children and adolescents are relatively rare. Despite the fact that some foreign studies have shown a similarity in brain volume heritability between adults and adolescents (the heritability of the whole brain volume is 0.89–0.91, the heritability of gray matter volume is 0.77–0.82^8, 9^), some other studies have seen significant differences in neonates compared with adults and adolescents^10^ (the heritability of gray matter volume is 0.56 in neonates). Wallace *et al*.^9^ carried out a further study on this topic, the results presented an increasing trend in terms of the heritability of gray matter volume from neonates to adolescents and a decreasing trend from adolescents to adults.

Currently, there is still a lack of observation on genetic and environmental impacts onbrain matter volume among children and adolescents, thus we hope to make a contribution to this area by taking advantage of our twins database.

## Discussion

Using twin study model to investigate the relationship between brain structure function and interaction of genetic and environment factors has become a hot topic in the field of psychiatry^11^. Classic twin study is based on the identical genetic background of MZ twins, assuming that the phenotype discrepancy is mainly caused by environment. In contrast, the probability of carrying identical genetic background for DZ twins is only 50%, whereby the heritability can be inferred. The advantage of SEM compared to conventional statistical approach is the ability to simultaneously process the latent variable and its index. Through SEM, a specific structural model will be developed to examine its correspondence to data. According to SEM, we can understand whether the relationship between different variables in the group remains unchanged. Multiple dependent variables can be simultaneously processed by SEM, allowing for the existence of measurement error among independent and dependent variables. Moreover, factor structures and their relationships can be estimated, providing the model with a greater elasticity and making it possible to estimate the degree of fitting of the whole model^12^.

This study shows that the cortex volume in most brain regions is mainly affected by genetic factors (0.70–0.89), no obvious differences were identified between two hemispheres (0.88/0.88). Peper, Wallace *et al*.^8, 9^ have conducted a similar study on gray matter volume in adults, the results showed that the heritability of the whole brain gray matter primarily distributed between 0.77–0.82. However, studies on gray matter volume of neonates showed that the heritability of left hemisphere is 0.71, while the right part is 0.44^10^. Similar studies showed that the heritability of the right hemisphere in adolescents is 0.19% larger than that of the left side^13^ on average. whereas in adults the heritability is 0.67 on the left and 0.64 on the right^6^, suggesting that the high heritability of gray matter could be related to human growth and development and the growing speed of right hemisphere is faster than the left side. Batouli S. A. *et al*.^14^ found that the genetic influences were still greater in the left hemisphere than in the right one as for elder twins, so we believe the left hemisphere tends to be more genetically affected than the right part.

The results of this study show that the heritability of the whole brain gray matter volume is 0.89. Previous studies have shown that the heritability of gray matter is increasing from neonate to child and adolescent period. Gilmore *et al*.^10^ found the heritability of gray matter volume is 0.56. Knickmeyer *et al*.^12^ found that the gray matter volume increases by approximately 149% from birth to 2 year old due to the rapid development of the neurons in related regions during that period. In contrast, the heritability of gray matter decreases from adolescent to adult9. Barre *et al*.^3^ found the heritability of gray matter in adults is 0.82, which might be resulted from the faster growth of white matter volume than gray matter volume. Batouli S. A. *et al*.^14^ found total brain volume (63%) and other volumetric measures were moderately to highly heritable in late life, and these genetic influences tended to decrease with age, suggesting a greater influence of environmental factors as age advanced.

The frontal lobe is found the most genetic. These findings support a strong coupling of variations in brain morphology and genetics^15^, particularly in the frontal cortex, which may underlie the high familial liability for some diseases that are associated with frontal cortical volume. (eg. schizophrenia)^16, 17^, in the bilateral cingulate region and bilateral medial temporal lobe region are less impacted by observing the heritability of regional brain gray matter. The bilateral cingulate region is mainly affected by individual-specific environmental effects, while the bilateral medial temporal lobe region is mainly influenced by both individual-specific environmental effects (E) and common environmental effects (C), and no genetic impact in the bilateral medial temporal lobe region also shows no obvious difference, the result is similar with what Kremen *et al*. found^13^, they found this phenomenon mainly appeared in entorhinal cortex of the medial temporal lobe and temporal pole, and region similar results were discovered in cingulated gyrus, considering these regions were less affected by genetic factors but influenced more by acquired environment. For example, the medial temporal lobe has been implicated in processing with memory and emotion. As such experience is likely to be specific for each individual, and not shared by members of a twin pair, unique environmental factors may be more important. Despite the use of the A/C/E model, we found the cortical volume of any region to show common environmental influences less than 55%, this may suggest importance of genes in the develop of the brain, but our small sample size may not have power to detect the C effect^18^, so a larger sample is necessary later.

## Conclusion

The heritability of brain gray matter of twins ranging in age from 12 to 18 in Chongqing district was investigated utilizing SEM and brain MRI technology. The results showed that the gray matter volume was mainly impacted by genetic factors in this period. Nevertheless, the impact of environment should not be neglected. Further studies are anticipated to make longitudinal comparisons by taking more factors into account.

## Methods and Participants

### Participants

Fifty-eight pairs of twins aged 12–18 in Chongqing were recruited based on demographic data from the school enrollment system of Chongqing education committee. Exclusion criteria: metallic foreign body, serious physical illness, mental illness or any other reasons for which the participants can’t afford brain MRI scan. After an informed consent form was signed by the legal guardian, and all the zygosity was collected, the research will be carried out. Monozygotic twin (MZ): 31 pairs, dizygotic twin (DZ): 27 pairs. ① age: 12–18 (15.2 ± 1.76), MZ: 15.34 ± 1.75, DZ: 15.07 ± 1.77; ② gender: male 51, female 65, MZ: male 26, female 36, DZ: male 25, female 29. This research was approved by Ethics Committee of Chongqing Medical University. The methods were carried out in accordance with the relevant guidelines. There was no statistically significant differences between the two groups in age and gender (P > 0.05).

## Tools

### Image acquisition

Images were acquired on a GE 3.0 T scanner (The First Affiliated Hospital of Chongqing Medical University, China), and the latest 8-channel head coil was used to improve the signal to noise ratio (SNR). 3D T1 images of all the participants were obtained by 3D gradient echo sequences. Time repetition = 6.2 ms, time echo = 2.8 ms, flip angle = 25°, slice thickness = 1.2 mm, one NEX, FOV = 24 cm * 24 cm, matrix = 256 * 256, and voxel size = 0.47 * 0.47 * 1 mm, a total of 166 axial slices were collected.

### Image processing

The Freesurfer software package^19–21^ was used to reconstruct the gray matter volume. The brain could be divided into gray and white matter, and the gray-white boundary was estimated. The Desikan-Killiany atlas^22^ wasused to divide the gray matter volume into 66 regions of interest (ROIs; 33 per hemisphere^21^). Then we calculated the volume of 12 cortical regions (bilateral frontal, parietal, occipital, lateral temporal, medial temporal and cingulated gyrus; see Table 1. For which Freesurfer parcellations are contained in each lobe) of interest based on the 66 regions. At last, the total gray matter volume was calculated as the sum of the areas of all ROIs.

**Table 1:** Correlation coefficient and model fitness of each brain region.

ROI	rMZ	rDZ	Parameter estimates with A, C, E influences on region Pvalue								
			A	95%CI	C	95%CI	E	95%CI	NO A	NO C	NO AC
L frontal	0.83	0.3	0.88	(0.63; 0.93)	0	(0; 0.24)	0.12	(0.07; 0.24)	<0.0001	1	<0.0001
R frontal	0.85	0.29	0.89	(0.65; 0.94)	0	(0; 0)	0.11	(0.06; 0.21)	<0.0001	1	<0.0001
L parietal	0.84	0.32	0.85	(0.53; 0.92)	0	(0; 0.3)	0.15	(0.08; 0.28)	<0.0001	1	<0.0001
R parietal	0.83	0.42	0.84	(0.39; 0.91)	0	(0; 0)	0.16	(0.09; 0.3)	<0.0001	1	<0.0001
L occipital	0.64	0.36	0.7	(0.11; 0.83)	0	(0; 0)	0.3	(0.17; 0.54)	0.02	1	<0.0001
R occipital	0.72	0.29	0.7	(0.21; 0.83)	0	(0; 0.4)	0.3	(0.17; 0.52)	0.01	1	<0.0001
L cingulate gyrus	0.48	0.29	0.48	(0; 0.68)	0	(0; 0.51)	0.52	(0.32; 0.84)	0.21	1	0.01
R cingulate gyrus	0.63	0.28	0.61	(0; 0.77)	0	(0; 0.51)	0.39	(0.23; 0.65)	0.06	1	<0.0001
L medial temporal	0.67	0.4	0.43	(0; 0.78)	0.21	(0; 0.67)	0.36	(0.22; 0.6)	0.19	0.57	<0.0001
R medial temporal	0.55	0.45	0.09	(0; 0.68)	0.42	(0; 0.66)	0.49	(0.3; 0.72)	0.79	0.24	<0.0001
L lateral temporal	0.67	0.5	0.72	(0.17; 0.87)	0.04	(0; 0.49)	0.24	(0.13; 0.44)	0.01	0.87	<0.0001
R lateral temporal	0.61	0.19	0.7	(0.3; 0.84)	0	(0; 0.27)	0.3	(0.16; 0.58)	0.01	1	<0.0001
Whole brain	0.86	0.39	0.89	(0.6; 0.94)	0	(0; 0.29)	0.11	(0.06; 0.20)	<0.0001	1	<0.0001
L hemisphere	0.85	0.37	0.88	(0.62; 0.94)	0	(0; 0)	0.12	(0.06; 0.19)	<0.0001	1	<0.0001
R hemisphere	0.85	0.36	0.88	(0.63; 0.94	0	(0; 0)	0.12	(0.06; 0.19)	<0.0001	1	<0.0001

The whole brain is divided into 12 brain region, A: additive genetic effects; C: common environmental effects; E: individual-specific environmental effects. CI: confidence interval. No A: CE model, hypothesis: there is no additive genetic effects; no C: AE model, hypothesis: there is no common environmental effects; no AC: E model, hypothesis: there is only individual-specific environmental effects.

### Zygosity identification

Zygosity (MZ or DZ) was determined by DNA from a blood sample. (instrument: Genetic Analyzer 3100AvantTM, Kit: AmpFLSTR® Identifiler® PCR Amplification). After all the participants and their legal guardian agreed, we collected blood sample and sent them to Mental Health Laboratory of West China Hospital of Sichuan to extract DNA. 15 short tandem repeat (STR) and Amelogenin were tested to determine zygosity. The reliability of 0.999 can be achieved using this genetic analysis.

### Statistical analysis

**Figure 1:**
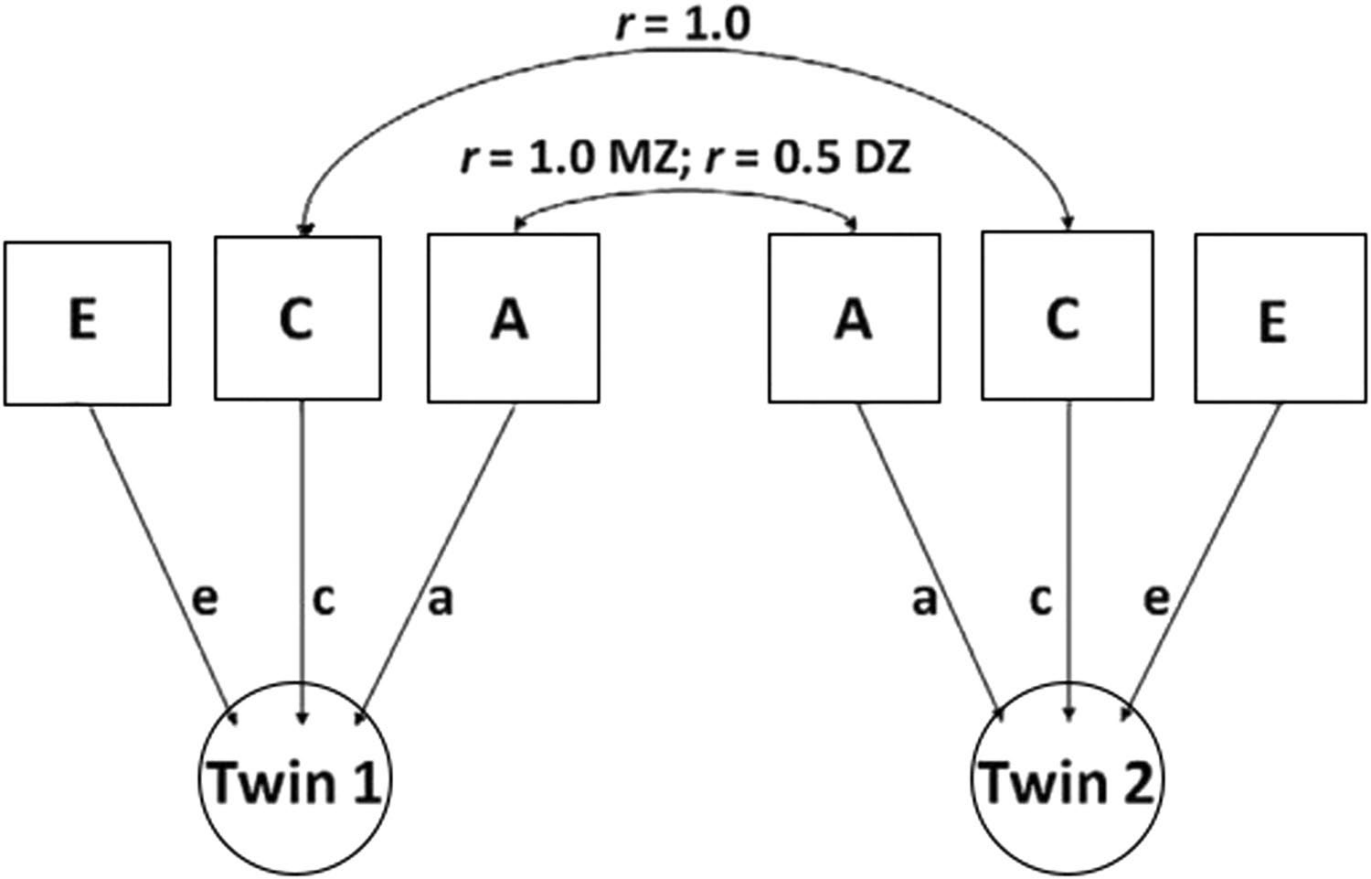
ACE model Univariate ACE model, A: additive genetic effects; C: common environmental effects; E: individual-specific environmental effects; a, c and e: path coefficient of A, C, and E. r: correlation coefficient; MZ: monozytic; DZ: dizygotic; Twin 1: elder one; Twin 2: youngerone.

SEM is widely used due to its unique advantages. It is able to build univariate, bivariate and multivariate model based on the relationship between the variables^23^. This study utilized univariate SEM model to analyze gray matter volume of each brain region. ACE model is commonly used in the twin study to evaluate the heritability by quantitatively calculating additive genetic effects (A) common environmental effects (C) and individual-specific environmental effects (E). C is what the twins experienced together, while E is what distinguished the difference of the twins. From Fig. 1 we find the correlation coefficient of genetic factor in MZ is 1, while the value is 0.5 in DZ; the correlation coefficient of common environmental effects is 1 in both MZ and DZ; and there is no correlation of individual-specific environmental effects in twins.

Based on the classic twin method, a rough estimation can be made on addictive genetic effects by correlation coefficient. When r MZ < r DZ, it is not suitable for genetic analysis; when rMZ > 2rDZ, it indicates the existence of genetic effect. Non-addictive genetic effects can be estimated and ADE model should be developed; when r DZ < rMZ < 2rDZ, common environmental effect is possibly existing, ACE model should be developed^11^. ACE/ADE models were compared with saturated model, no differences were found between them (P > 0.05). On the other hand, the impact of A + D in ADE model was comparable to that of A in ACE model. As a result, we only used ACE model to simplify the comparison of heritability in each brain region^24^.

**Figure 2:**
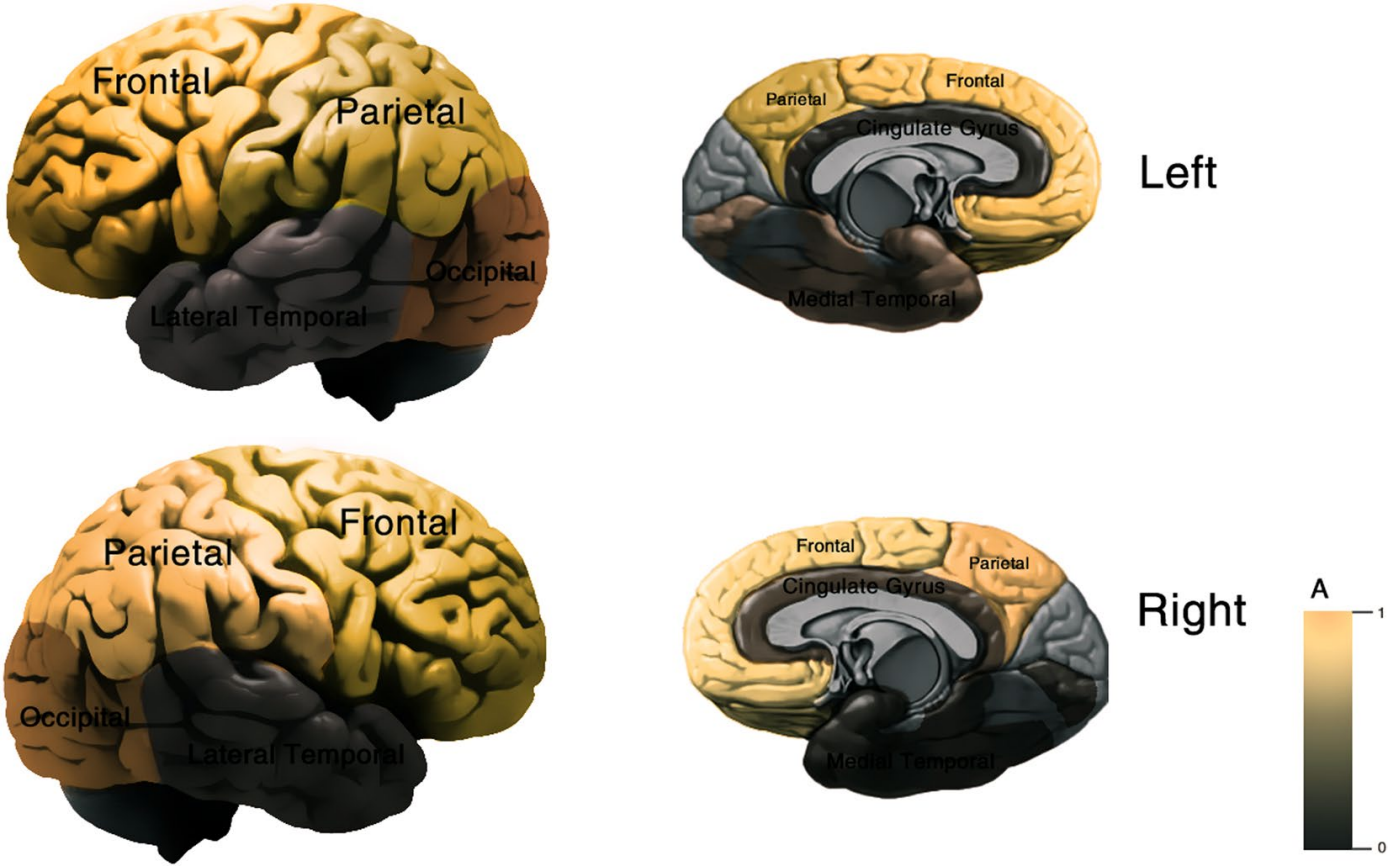
Additive genetic effects in both hemispheres. We mainly divided the brain into 6 lobes which is frontal/parietal/occipital/medial temporal/cingulate gyrus/lateral temporal by different colors. “A” (the color key) means heritability represented certain brain regions, respectively.

All the data were statistically processed by spss20.0 statistical software package Open-mx package was utilized to fit SEM genetic model. The results are shown in Table 1 and Fig. 2.

## Results

Correlation coefficient and model fitness of each brain region (Table 1 and Fig. 2). From the results: r MZ > r DZ in all the regions. From 12–18, the whole brain gray matter volume was mainly impacted by genetic factors (A: 0.89), and no obvious differences were found between the two hemisphere (0.88/0.88). The gray matter volume was mostly affected by genetics in the frontal, parietal, occipital and lateral temporal lobe, while in cingulate and medial temporal of gray matter volume, it was jointly affected by genetic and environment factors^25^ (A: 0.09–0.61, C: 0–0.42, E: 0.36–0.52).
